# Association between chronic pain severity, falls, frailty and perceived health in older adults at risk of falls

**DOI:** 10.1186/s40001-025-03304-w

**Published:** 2025-10-31

**Authors:** Denishkrshna Anbarasan, Reshma Aziz Merchant

**Affiliations:** 1 Department of Medicine, Yong Loo Lin School of Medicine, National University of Singapore, Singapore, Singapore 119228; 2 Division of Geriatric Medicine, Department of Medicine, National University Hospital, 1E Kent Ridge Road, Singapore, 119228 Singapore

**Keywords:** Chronic pain, Falls risk, Older adults, Quality of life, Frailty, Back pain

## Abstract

**Background:**

Chronic pain, defined as pain persisting for ≥ 3 months, is associated with frailty, falls, and reduced quality of life. Falls remain a major cause of morbidity and mortality in older adults, yet the value of integrating pain severity into falls risk assessment is underexplored. This study examined associations between chronic pain severity and subsequent falls, frailty, physical performance, and perceived health in older adults at risk of falls.

**Methods:**

In this cross-sectional study, baseline data from 143 community-dwelling adults aged ≥ 60 years at risk of falls were analysed. Participants were recruited from community and primary care centres in Singapore. Pain severity was assessed using the Wong–Baker Faces Pain Rating Scale (0–10) and classified as no pain, mild pain (< 3), or at least moderate pain (≥ 3). Data on demographics, frailty, sarcopenia (SARC-F ≥ 2), nutrition, cognition, fear of falling, and perceived health (EuroQol Visual Analogue Scale [EQ-VAS] and EuroQoL 5-Dimensions [EQ-5D]) were collected. Physical performance tests included handgrip strength, gait speed, 5-times sit-to-stand, and Timed-Up-and-Go (TUG). Logistic and linear regression models examined associations between pain severity and outcomes, adjusting for demographic and clinical covariates.

**Results:**

Chronic pain was prevalent in 37.1%. Compared with no pain, at least moderate pain was associated with higher odds of future falls (adjusted odds ratio (aOR) 3.54, 95% CI 1.53–8.19), moderate/high falls risk (aOR 4.78, 95% CI 1.65–10.77), frailty (aOR 4.17, 95% CI 1.42–8.26), sarcopenia (aOR 4.99, 95% CI 1.63–7.28), slower gait speed (aOR 3.87, 95% CI 1.18–8.67), longer TUG (aOR 4.52, 95% CI 1.36–10.01), and poor physical performance (aOR 12.50, 95% CI 3.94–17.17). Pain severity was associated with EQ-VAS (β = − 4.07, 95% CI − 7.67 to − 1.47) and EQ-5D index (β = − 0.11, 95% CI − 0.15 to − 0.07).

**Conclusion:**

Higher chronic pain severity was associated with future falls, frailty, poor physical performance and lower perceived health in at-risk older adults. Incorporating pain severity assessment into falls risk stratification could support earlier, targeted interventions to prevent injurious falls. Longitudinal studies are needed to determine the causal impact of pain management on falls, frailty, and quality of life.

**Clinical relevance:**

Chronic pain in older adults is significantly associated with an increased risk of future falls, frailty, poor physical performance, and lower perceived health, highlighting the importance of evaluating chronic pain in fall risk assessments and vice versa. Implementing targeted prevention measures for individuals with chronic pain can potentially mitigate the risk of falls and improve overall health outcomes in this population.

## Background

With the global population aging, the prevalence of noncommunicable diseases such as obesity, cardiometabolic diseases, and frailty is expected to rise. These conditions are well-documented risk factors for falls, poor physical performance, and chronic pain, and their increasing burden will place substantial strain on health and social care systems. In the ICD-11 classification, the World Health Organization defines chronic pain as pain persisting or recurring for more than three months and is one of the most critical health concerns of the decade [1]. Pain expression can be influenced by resilience, psychosocial factors, functional ability, and underlying comorbidities [2]. The prevalence of chronic pain varies between 35.0% and 51.3%, with moderate-to-severe pain ranging between 10.4% and 14.3% [3]. Age is one of the most important risk factors, with up to 62% of individuals over 75 years old experiencing chronic pain [3]. Chronic pain is associated with injurious falls, frailty, cognitive impairment, malnutrition, quality of life, disability, sleep disturbance, and mortality [3–8].

Falls are a leading cause of accidental deaths among older adults, significantly contributing to morbidity, mortality, and healthcare costs. In the United States, approximately 14 million or one in four older adults aged 65 years and above report a fall each year, resulting in an estimated 9 million fall-related injuries [9]. Healthcare expenditures for fatal and nonfatal falls among this population reached an estimated $50 billion annually in 2015 [10], and age-adjusted fall-related death rates rose by 41%, from 55.3 per 100,000 in 2012 to 78.0 per 100,000 in 2021 [11]. Older adults with chronic multisite severe pain have greater declines in mobility and increased risk of falls, especially injurious falls [12]. Falls in these individuals could be due to lower limb weakness, poor mobility and balance, adverse effects of analgesia and change in gait dynamics, which affect postural control [5, 13].

Frailty is a dynamic state of reduced physiological reserve that predisposes the individual to adverse outcomes when exposed to stressors [14]. Chronic pain, falls and frailty share a triangular relationship, with shared pathophysiological pathways, such as chronic inflammation and mitochondrial dysfunction [15]. Mobility and chronic pain share the same central nervous system pathway and several brain regions such as the anterior cingulate cortex, insular cortex, prefrontal cortex and thalamus [16]. Chronic pain in older adults can disrupt homeostatic mechanisms, compromise functional reserve and accelerate frailty progression described as “pain homeostenosis” by Shega et al. [17, 18].

Despite extensive research on fall risk factors, the incidence of falls and injurious falls remains unchanged. Therefore, a more comprehensive approach to understanding and mitigating fall risk among older adults is necessary. Although there is evidence indicating that chronic pain can be managed with exercise, pharmacologic treatments, and cognitive-behavioral therapies, current management of chronic pain remains reactive. Long-term solutions are often not implemented, with chronic pain frequently attributed to aging [2]. While the association of chronic pain with falls is known, few studies have examined the association between chronic pain severity and frailty, poor physical performance, sarcopenia, future falls and perceived health simultaneously in an at-risk older population. Understanding these associations will facilitate the implementation of secondary and tertiary prevention measures for individuals with falls risk. This study aims to determine the prevalence of chronic pain in older adults with falls risk, and its association with subsequent falls, frailty, gait speed, poor physical performance, and quality of life.

## Methods

### Study participants

One hundred and fifty-four participants aged 60 years and above were recruited through convenience sampling from community and primary care centres in Singapore as part of a falls prevention intervention trial. After excluding ten participants who experienced new pain in the last three months, one hundred and forty-three participants were included in the final analysis. Older adults aged 60 years and above were eligible if they were community-dwelling, ambulant (with or without a walking aid), had experienced at least one fall or near fall in the past 12 months, and were able to provide informed consent and follow study instructions. Individuals were excluded if they were nursing home residents, had a pacemaker or defibrillator, had underlying psychiatric conditions, or were unable to ambulate due to severe musculoskeletal pain, neurological disease, or other physical impairments that would preclude safe participation in physical performance testing and exercise interventions. This study conformed to the principles of the Declaration of Helsinki and was approved by The National Healthcare Group Domain Specific Review Board (Reference: 2019/00650). Informed consent was obtained from all participants involved in the study.

### Pain

Chronic pain was defined as having pain for at least three months [1]. The Wong–Baker Faces Pain Rating Scale (WBFS) was used to evaluate participants’ pain severity [9]. WBFS was selected for its simplicity, visual accessibility, and validated use across culturally diverse populations. Its pictorial format minimizes reliance on language, making it particularly effective in culturally diverse populations like Singapore and among individuals with limited literacy or cognitive impairment. Participants were presented with a visual scale of faces with numbers ranging from 0 (no pain) to 10 (worst pain) and were asked to rate their pain. Participants were also asked to indicate at most two sites where they experienced pain. Those who did not experience any pain were included in the ‘No Pain’ group, while those with pain were further grouped based on the WBFS cut-off value of mild pain (< 3) or at least moderate pain (≥ 3).

### Covariates

Trained study team members gathered information with regard to participants’ demographic covariates including gender, ethnicity, body mass index (BMI), years of education, multimorbidity (≥ 2 chronic conditions), polypharmacy (≥ 5 medications), and nutritional risk (Simplified Nutritional Appetite Questionnaire score ≤ 14). Physical activity was assessed using the Rapid Physical Assessment (RAPA) [19]. Instrumental activities of daily living (IADL) were assessed using Lawton and Brody’s IADL questionnaire [20]. Activities of daily living (ADL) were evaluated using Katz’s ADL questionnaire [21]. Simplified Nutritional Appetite Questionnaire (SNAQ) was used to assess nutrition. With a total score ranging from 4 to 20, a score ≤ 14 was considered to be at risk of malnutrition [22]. Depression was assessed using the 15-item Geriatric Depression Scale (GDS). With a maximum score of 15, a cut-off of ≥ 5 was used to define depression [23]. Participants’ life-space mobility was assessed using the validated University of Alabama at Birmingham Life-Space Assessment questionnaire. Life-space mobility index is a measure of the ability of an individual to move within their own homes, across the environment or region, with greater scores indicating higher mobility [24, 25].

### Falls, cognition, frailty, sarcopenia and fear of falling

Cognition was evaluated using the Montreal Cognitive Assessment (MoCA), and cognitive impairment was defined as having MoCA scores < 26 [26]. The 5-item FRAIL scale was used to assess participants’ frailty status. Those with a score of 0 were considered robust, 1–2 as pre-frail and ≥ 3 as frail [14]. The SARC-F questionnaire was used to assess participants’ risk of sarcopenia, with a cut-off score of ≥ 2 to improve sensitivity to detect sarcopenia [27, 28]. The Falls Efficacy Scale (FES), a 19-item survey, was used to evaluate participants’ concerns over falling, with total scores ranging from 16 to 64, higher scores associated with greater concerns [29]. Participants’ baseline falls risk was assessed using the Falls Risk for Older People in the Community Assessment (FROP-Com) tool, a comprehensive falls risk assessment tool developed by the National Ageing Research Institute Australia [30]. Those with a FROP-Com score ≤ 11 were considered to have low falls risk, 12–18 as moderate risk, while ≥ 19 as high risk.

### Perceived health and quality of life

Perceived health was assessed using the EuroQoL Visual Analogue Scale (EQ-VAS), while health-related quality of life was measured using the EuroQoL-5 Dimensions (EQ-5D) questionnaire. The EQ-5D responses were converted to a single index score using the Thai population value set, with scores ranging from 0 to 1, where a score of 0 indicates death and 1 indicates perfect health [31].

### Physical performance

Physical performance measures comprised maximum handgrip strength (HGS), gait speed, Short Physical Performance Battery (SPPB), and the Timed-Up-and-Go (TUG) test. HGS was measured using a Jamar hand dynamometer on the dominant hand with the participant in a seated position and the elbow flexed at 90°. Low HGS was defined as < 28 kg for males and < 18 kg for females [32]. The SPPB, with 3 components—balance, gait speed and 5 × sit-to-stand (STS) timing, has a maximum score of 12 points (4 per component). Gait speed < 1.0 m/s was considered slow gait speed [32]. Participants with either slow gait speed, 5 × STS ≥ 12 s, or SPPB total score ≤ 9 were considered to have poor physical performance [32]. For the TUG test, participants were instructed to walk 3 m to and fro a seated position. Time was recorded from the point the participants stood up until they sat back down [33].

### Statistical analysis

**Fig. 1:**
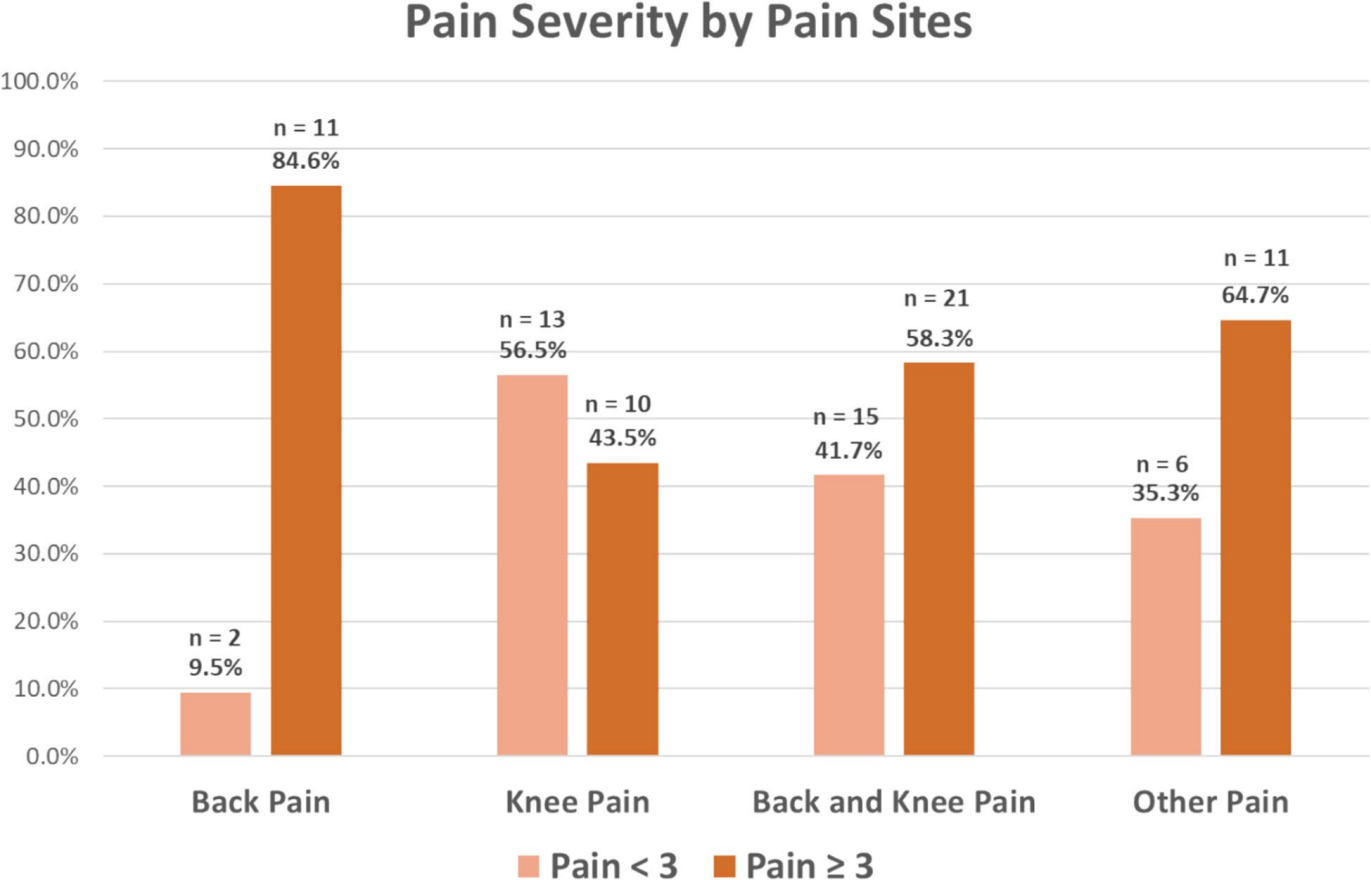
Distribution of pain severity across back, knee and other sites

IBM SPSS Version 29.0 was used for data analysis with statistical significance set at 2-sided 5%. Categorical variables were presented as frequencies with percentages, while continuous variables were presented as mean ± standard deviation. χ2 test was used for significance testing of categorical variables. For continuous variables, the normality assumption was tested using the Shapiro–Wilk test. Significance testing for normally distributed variables was carried out using one-way ANOVA. The Kruskal–Wallis test was used when continuous variables were not normally distributed. Post hoc tests were Bonferroni corrected. The prevalence of either back pain, knee pain, back and/or knee pain, and pain in other sites, by pain severity, is tabulated in Fig. 1.

The primary exposure variable was chronic pain severity, categorized as no pain, mild pain, or at least moderate pain. The primary outcome was self-reported falls in the subsequent three months. Associations between pain severity and baseline characteristics, including frailty status, physical performance measures (TUG, gait speed, handgrip strength), sarcopenia, and falls risk, were also examined. Logistic regression models were used for binary variables (e.g., future falls, frailty status), ordinal logistic regression for ordered categorical variables (e.g., FES tertiles, FROP-Com categories), and linear regression for continuous variables (e.g., EQ-VAS, EQ-5D index). All models used pain severity as the independent variable, with no pain as the reference group. The models were adjusted for potential confounders, including age, gender, ethnicity, body mass index (BMI), education, multimorbidity, nutrition, and polypharmacy. Odds ratios (ORs) or β coefficients with 95% confidence intervals (CIs) were reported, with statistical significance set at p < 0.05.

## Results

### Participant characteristics and demographics

Of the 143 participants enrolled in this study, chronic pain was prevalent in 37.1% [53] participants. WBFS rating ranged from 2 to 6 with a median of 3. Amongst them, 14.7% [21] had mild pain and 22.4% [32] had at least moderate pain (Table 1). The most common pain sites were back (9.0%), knee (16%) and combined back and knee (25.2%). At least moderate pain was prevalent in 84.6% [11] of the back pain group, 38.5% [10] of the knee pain group and 58.3% [21] of the combined back and knee pain group (Fig. 1).

**Table 1:** Baseline characteristics

	Bothered by pain for at least 3 months				
	Total	No pain	Mild pain	At least moderate pain	P-value
	n = 143	n = 90 (62.9%)	n = 21 (14.7%)	n = 32 (22.4%)	
Demographics					
Gender					0.238
Male	55 (38.5)	35 (38.9)	5 (23.8)	15 (46.9)	
Female	88 (61.5)	55 (61.1)	16 (76.2)	17 (53.1)	
Age (years)	74.83 ± 7.78	75.57 ± 7.91	75.05 ± 6.14	72.63 ± 8.19	0.184
Ethnicity					0.163
Chinese	103 (72.0)	66 (73.3)	17 (81.0)	20 (62.5)	
Malay	17 (11.9)	13 (14.4)	2 (9.5)	2 (6.3)	
Indian	20 (14.0)	10 (11.1)	2 (9.5)	8 (25.0)	
Others	3 (2.1)	1 (1.1)	0 (0.0)	2 (6.3)	
BMI (kg/m^2^)	24.43 ± 4.38	23.62 ± 4.23^a^	25.34 ± 4.02	26.29 ± 4.58^a^	0.008
Education (years)	5.50 ± 4.43	4.91 ± 4.34	5.73 ± 4.01	7.03 ± 4.71	0.081
Multimorbidity	93 (65.0)	55 (61.1)	16 (76.2)	22 (68.8)	0.124
Hypertension	84 (58.7)	49 (54.4)	15 (71.4)	20 (62.5)	0.240
Hyperlipidaemia	79 (55.2)	46 (51.1)	14 (66.7)	19 (59.4)	0.785
Diabetes	38 (26.6)	24 (26.7)	7 (33.3)	7 (21.9)	0.333
Osteoporosis	9 (6.3)	7 (7.8)	0 (0.0)	2 (6.3)	0.415
Previous fracture	26 (18.2)	12 (13.3)	5 (23.8)	9 (28.1)	0.183
Polypharmacy	5 (3.5)	2 (2.2)	2 (9.5)	1 (3.1)	0.229
Perceived Health (EQ-VAS)	68.42 ± 16.59	71.82 ± 15.49^a,b^	62.43 ± 21.1^a^	63.02 14.28^b^	0.007
EQ-5D Index Score	0.79 ± 0.19	0.9 ± 0.2^a,b^	0.7 ± 0.2^a^	0.6 ± 0.2^b^	< 0.001
Physical Activity (RAPA)	3.30 ± 1.62	3.37 ± 1.60	3.05 ± 1.23	3.28 ± 1.87	0.730
At least 1 IADL impairment	55 (38.5)	34 (37.8)	7 (33.3)	14 (43.8)	0.730
At least 1 ADL impairment	27 (18.9)	16 (17.8)	5 (23.8)	6 (18.8)	0.817
Frailty status					0.020
Robust	59 (41.3)	44 (48.9)	8 (38.1)	7 (21.9)	
At least pre-frail	84 (58.7)	46 (51.1)	13 (61.9)	25 (78.1)	
SARC-F ≥ 2	70 (49.0)	34 (37.8)	15 (65.2)	21 (70.0)	0.002
MoCA	22.56 ± 6.05	21.76 ± 6.65	23.20 ± 5.06	24.34 ± 4.40	0.104
Depression	48 (33.6)	31 (34.4)	7 (33.3)	10 (31.3)	0.947
Nutritional status (SNAQ)					0.010
Normal nutritional status	99 (69.2)	70 (77.8)	13 (61.9)	16 (50.0)	
At risk of malnourishment	44 (30.8)	20 (22.2)	8 (38.1)	16 (50.0)	
Life Space Assessment	63.20 ± 23.67	63.18 ± 24.41	56.90 ± 20.56	67.20 ± 23.17	0.314
Falls					
Falls in past 3 months					0.013
1 Fall	33 (23.1)	19 (21.1)	3 (14.3)	11 (34.4)	
≥ 2 Falls	16 (11.2)	5 (5.6)	4 (19.0)	7 (21.9)	
Falls Efficacy Scale Total	21.73 ± 6.73	20.61 ± 5.97^a,b^	23.55 ± 5.64^a^	23.75 ± 8.63^b^	0.032
Lower tertiles	95 (66.4)	68 (75.6)	12 (57.1)	15 (46.9)	0.004
Highest tertile	48 (33.6)	22 (24.4)	9 (42.9)	17 (53.1)	
Falls risk (FROP-Com)					0.017
Low	81 (56.6)	61 (67.8)	9 (42.9)	11 (34.4)	
Moderate/high	62 (43.4)	29 (32.2)	12 (51.1)	21 (65.6)	
Physical performance					
Handgrip strength (kg)	18.93 ± 7.05	19.01 ± 7.15	17.87 ± 7.06	19.38 ± 6.93	0.736
Low handgrip strength^1^	100 (69.9)	63 (70.0)	14 (66.7)	23 (71.9)	0.921
Gait speed (m/s)	0.91 ± 0.34	0.93 ± 0.36	0.85 ± 0.39	0.87 ± 0.25	0.474
Slow gait speed	92 (64.3)	51 (56.7)	16 (76.2)	25 (78.1)	0.063
5 × sit-to-stand time (s)	15.45 ± 5.47	15.09 ± 5.61	15.70 ± 4.40	16.51 ± 5.83	0.540
Total SPPB Score	8.67 ± 2.68	8.98 ± 2.71	8.24 ± 2.49	8.09 ± 2.64	0.204
TUG (s)	13.97 ± 8.95	13.78 ± 9.62	13.71 ± 5.51	14.65 ± 9.05	0.891
Poor physical performance	118 (82.5)	67 (74.4)	19 (90.5)	32 (100.0)	0.003

Values presented as n (%) or mean ± SD; bold indicates significance (p < 0.05); ^ab^ Values with common superscript are significantly different after Bonferroni correction;

*BMI* body mass index; ADL, Activities of Daily Living, *MoCA* Montreal Cognitive Assessment; *SPPB* Short Physical Performance Battery; Timed-up-and-go (TUG)^1^ Adjusted for gender

Table 1 shows baseline demographics where 61.5% (88) were females and the mean age was 74.8 ± 7.8 years. BMI was highest in those with at least moderate pain (26.3 kg/m^2^), followed by mild pain (25.3 kg/m^2^) and no pain (23.6 kg/m^2^). More than three-quarters (78.1%) of those with moderate pain were at least pre-frail, compared with almost two-thirds (61.9%) of those with mild pain and one-half (51.1%) with no pain. More than two-thirds of those with at least moderate pain (70.0%) and mild pain (65.2%) scored ≥ 2 for SARC-F as compared to those with no pain (37.8%). Those with at least moderate pain were at higher risk of malnutrition (50.0%), compared with those with mild pain (38.1%) or no pain (22.2%).

### Falls and fear of falling

 One-third (33.3%) with mild pain and more than half (56.3%) with at least moderate pain experienced at least one fall in the subsequent 3 months, compared to slightly more than one-quarter (26.7%) of the no pain group. Participants with pain had a greater FES total (23.8 ± 8.6 vs. 23.6 ± 5.6 vs. 20.6 ± 6.0) than those without pain. When FES total scores were split into tertiles, those with at least moderate pain (53.1%) had the highest proportion in the highest tertile, followed by mild pain (42.9%) and no pain (24.4%). Almost two-thirds (65.6%) of those at moderate to high falls risk on FROP-Com had at least moderate pain, and half (51.1%) mild pain.

### Physical performance

The proportion of participants with poor physical performance was significantly greater in those with at least moderate pain, followed by mild pain and no pain (100.0%, 90.5%, and 74.4%, respectively) (Table 1).

### Perceived health and quality of life

**Fig. 2:**
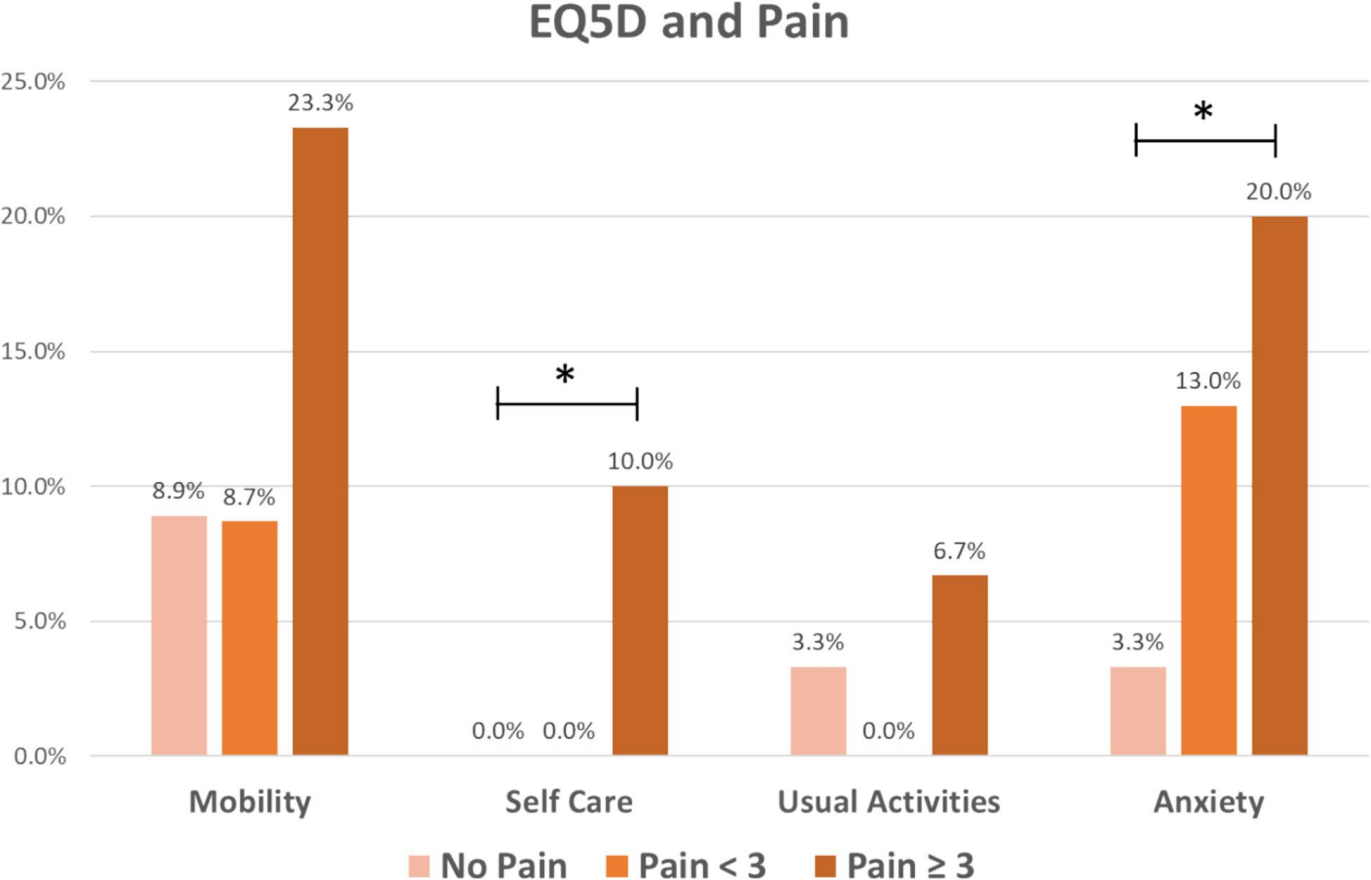
Distribution of EQ5D domains with at least moderate difficulty (score ≥ 3). * indicates significant difference (p < 0.05)

Perceived health was significantly lower in mild pain (62.4) and at least moderate pain (63.0) compared with no pain (71.8). The distribution of respective EQ-5D domains with at least moderate difficulty (score ≥ 3) is reflected in Fig. 2. Those with at least moderate pain had the highest proportion of individuals facing at least moderate difficulty in the EQ5D self-care domain (10.0% vs. 0% vs. 0%) and were at least moderately anxious or depressed (20.0% vs. 13.0% vs. 3.3%) (Fig. 2). The EQ-5D health index was significantly higher in no pain (0.9) compared with mild pain (0.7) and at least moderate pain (0.6).

### Association between chronic pain severity, frailty, falls, physical performance and perceived health

When adjusted for age, gender, ethnicity, BMI, education, multimorbidity, nutrition and using no pain as a reference, having at least moderate pain was significantly associated with increased odds of reporting ≥ 1 fall in the subsequent 3 months (aOR 3.49, 95% CI 1.25 to 9.75, p = 0.017), and highest FES tertile (aOR 3.76, 95% CI 1.21–7.68, p = 0.022). Chronic pain, regardless of severity, was associated with moderate/high falls risk (mild pain: aOR 3.32, 95% CI 1.09 to 10.13, p = 0.035 and at least moderate pain: aOR 4.78, 95% CI 1.65 to 10.77, p = 0.004) (Table 2).

**Table 2:** Association of pain severity with falls and functional measures (reference group: no pain)

	Mild pain		At least moderate pain	
	Unadjusted β (95% CI); p-value	Adjusted^#^ β (95% CI); p-value	Unadjusted β (95% CI); p-value	Adjusted^#^ β (95% CI); p-value
Falls ≥ 1 in the past 3 months	1.38 (0.50–3.82); p = 0.541	1.29 (0.41–4.08); p = 0.662	3.54 (1.53–8.19); p = 0.003	3.49 (1.25–9.75); p = 0.017
Falls efficacy scale				
Lower tertile	Reference	Reference	Reference	Reference
Highest tertile	2.53 (0.93–6.90); p = 0.070	1.52 (0.43–5.35); p = 0.517	3.50 (1.51—8.15); p = 0.004	3.76 (1.21–7.68); p = 0.022
Falls risk (FROP-Com)				
Low	Reference	Reference	Reference	Reference
Moderate/high	2.81 (1.06–7.40); p = 0.037	3.32 (1.09–10.13); p = 0.035	4.02 (1.71–9.42); p = 0.001	4.78 (1.65–10.77); p = 0.004
Frailty status				
Robust	Reference	Reference	Reference	Reference
At least pre-frail	1.49 (0.59–3.79); p = 0.454	1.46 (0.50–4.28); p = 0.489	3.83 (1.43–10.25); p = 0.008	4.17 (1.42–8.26); p = 0.009
Sarcopenia (SARC-F ≥ 2)	2.68 (.81–7.12); p = 0.293	2.28 (0.73–7.06); p = 0.155	4.21 (1.75–8.15); p = 0.001	4.99 (1.63–7.28); p = 0.005
Cognitive impairment	0.93 (0.35–2.47); p = 0.889	1.37 (0.37–5.01); p = 0.636	0.56 (0.24–1.28); p = 0.171	0.77 (0.25–2.40); p = 0.647
Low handgrip strength	0.86 (0.31–2.36); p = 0.766	1.24 (0.39–3.99); p = 0.715	1.10 (0.45–2.68); p = 0.842	2.21 (0.70–6.92); p = 0.175
Slow gait speed	2.32 (0.78–6.90); p = 0.130	2.44 (0.72–8.24); p = 0.151	2.59 (1.01–6.63); p = 0.047	3.87 (1.18–8.67); p = 0.025
Slow TUG	1.69 (0.62–4.65); p = 0.308	2.03 (0.60–6.92): p = 0.256	2.23 (0.92–5.39); p = 0.076	4.52 (1.36–10.01); p = 0.014
Poor physical performance	4.81 (1.06–11.77); p = 0.042	8.29 (1.56–14.22); p = 0.013	12.02 (1.56–19.72); p = 0.017	12.50 (3.94–17.17); p = 0.002

Reference group: no pain; values presented as odds ratio (95% confidence interval); bold indicates significance (p < 0.05); ^#^ adjusted for age, gender, ethnicity, body mass index, education, multimorbidity and nutrition. Timed-up-and-go (TUG)

Participants with at least moderate pain had higher odds of being classified as pre-frail or frail (aOR 4.17, 95% CI 1.42 to 8.26, p = 0.009), SARC-F ≥ 2 (aOR 4.99, 95% CI 1.63–7.28, p = 0.005), slow gait speed (aOR 3.87, 95% CI 1.18 to 8,67, p = 0.025), and slow TUG (aOR 4.52, 95% CI 1.36 to 10.01, p = 0.014). Increasing pain intensity was significantly associated with lower perceived health scores defined by EQ-VAS (β = − 4.07, 95% CI − 7.67—− 1.47, p = 0.027) and EQ5D index (β =− 0.11, 95% CI − 0.15—−0.07, p < 0.001) (Table 3).

**Table 3:** Linear association of pain with health status

	Unadjusted β coefficient (95% CI); p-value	Adjusted^#^ β coefficient (95% CI); p-value
EQ-VAS	− 5.13 (− 8.13—− 0.94); p = 0.002	− 4.07 (− 7.67—− 1.47); p = 0.027
EQ5D Index	− 0.12 (− 0.17—− 0.09); p < 0.001	− 0.11 (− 0.15—− 0.07); p < 0.001

Values presented as β coefficient (95% confidence interval); bold indicates significance (p < 0.05); ^#^ adjusted for age, gender, ethnicity, body mass index, education, multimorbidity, and nutrition

## Discussion

Our study showed that chronic pain, regardless of severity is prevalent in more than one-third of older adults at risk of falls. The majority of them had at least moderate pain with top sites being back and/or knee pain. Those with at least moderate pain had higher BMI, increased risk of malnutrition, frailty, falls, fear of falling, and poor physical performance. At least moderate pain was associated with a higher prevalence of falls reported in the subsequent 3 months. It was also associated with higher odds of being classified as frail or pre-frail, slower gait speed, longer TUG and poor physical performance. The connection between chronic pain and falls is well-documented in the community. A most recent publication likewise reported that both increasing pain intensity and multisite pain were independently associated with almost twice the risk of falls [34]. Similarly, Nagashima et al. [35] showed gender differences in Japanese people 40–74 years old where the association of chronic knee pain with recurrent falls was significant in women and chronic low back pain in men. Multisite pain has also been associated with a 57% increased risk for injurious falls [4]. The World Falls Guideline recommends the inclusion of pain assessment as part of a multifactorial falls risk evaluation, although this recommendation is based on GRADE E evidence [36].

The observed association between chronic pain and falls could be partially attributed to musculoskeletal weaknesses, particularly in the lower back and lower limb muscles. In addition, multiple brain regions such as the anterior cingulate cortex, prefrontal cortex, and thalamus, which are key regions implicated in pain perception and emotional processing, play critical roles in mediating chronic pain and influencing gait speed through their impact on motor control, coordination, and sensory integration [37]. Poor STS performance could be due to pain-related limitations or weakness of the rectus femoris or gastrocnemius, which is prevalent in individuals with osteoarthritis of the knee. In addition, weak abdominal and/or pelvic floor muscles can lead to imbalance in the core and an increase in the load of the lower back, with an impact on the erector spinae and gluteal muscle affecting mobility, balance, and causing falls [38, 39]. Pain may cause altered kinematics between the spine, pelvis, and lower limb, affecting coordination, variability in movement patterns, and altered activation of different muscle groups [40, 41]. Overactivation and inefficient movements can lead to increased fatigue resulting in a downward spiral of fatigue and weakness [42]. Chronic lower back pain has been linked to weakened abdominal and trunk muscles, while anterior knee pain has been associated with quadriceps femoris strength deficits [43, 44].

Most of the participants with pain in our study had both back and/or knee pain. One of the most common sites is the lower back. In 2023, the World Health Organization released a guideline on the management of chronic low back pain in primary and community care settings [45, 46]. Tse et al. [47] reported an increased association of low back pain or severe lower limb pain with recurrent falls and falls-related injury in females. More than one-quarter of participants with at least moderate pain in our study had a previous history of fracture. Chronic low back pain and low step counts have been associated with social frailty [48]. However, there was no significant difference in the Life Space Assessment Scores, and no significant difference in the prevalence of depression between groups.

More than three-quarters of our participants with at least moderate chronic pain were either pre-frail or frail. Frailty is independently associated with adverse outcomes such as falls, poor perceived health, and mortality, and it is possibly reversible before the onset of disability [14]. A systematic review reported an overall pooled prevalence of 18% for frailty and 43% for pre-frailty in older adults with chronic pain, which was similar to our participants with mild chronic pain [49]. In the same study, half of frail and one-third of pre-frail older adults reported chronic pain [49]. Those with at least moderate pain in our study had four times higher odds of being pre-frail or frail. A systematic review by Lin et al. [42], which included 10 studies, showed that non-frail participants with chronic pain were 1.85 times more likely to develop frailty over 5.8 years. The management of older adults with chronic pain and frailty requires a personalized approach incorporating multidisciplinary and adopting a multi-domain approach, which includes nutrition and exercise [14]. Frail individuals may be particularly susceptible to the adverse effects of analgesics. Additionally, cognitive impairment and dementia are prevalent in frail individuals, which may lead to a delay in diagnosis and management, further impacting physical performance [50]. To date, there are no specific guidelines on the management of chronic pain in frailty and its impact on long-term outcomes.

Unlike Rouch et al. [51], our study did not demonstrate a longitudinal association between chronic pain and cognition. Similarly, one-third of the participants had underlying depression with no significant difference between the groups. This could be due to our study being cross-sectional in nature, involving a high-risk population. Participants with pain had significantly higher BMI. Indeed, obesity has a significant association with chronic pain independent of insulin resistance and/or inflammation, osteoarthritis, or neuropathy [52]. The prevalence of individuals at risk of malnutrition was significantly higher in the group with at least moderate pain. Chronic pain, physical function, frailty, and nutrition appear to be interlinked through shared pathophysiological mechanisms, including systemic inflammation, obesity, dysregulation of the microbiota–gut–brain axis, impaired glucose metabolism, and altered lipid metabolism (53).

This study is not void of limitations. First and foremost, the small sample size limited our ability to analyse chronic pain based on various sites and gender. This limitation may have resulted in an underestimation of the potential differential impacts of pain site and gender on outcomes. Second, the cross-sectional nature of this study involving individuals with falls limits the ability to infer causality and the applicability of the findings at the population level. Third, we have no information on analgesia use for pain, which itself may be a risk factor for falls or malnutrition. Fourth, we had no information on the exact duration of pain or if the onset of pain was after the fall or injuries. Fifth, information on falls, pain, and demographics may be affected by recall bias. Key physiological measures such as proprioception and lower limb strength were not assessed directly, limiting our understanding of the mechanism linking chronic pain to falls. Lastly, limiting enrolment to ambulant older adults and excluding individuals with mobility limitation which could be due to pain amongst other reasons could potentially bias toward those less severely affected. This may under-represent the true burden of chronic pain on falls risk in non-ambulatory older adults. Future studies should investigate this very high subgroup with personalized recommendations. Nevertheless, this study contributes valuable insights into the relationship between chronic pain, falls, frailty, and physical performance among at risk community-dwelling older adults. The findings reinforce the need for routine pain assessments as part of fall prevention strategies, multifactorial risk assessments for falls in those with chronic pain, and assessment for frailty in those with chronic pain and falls.

Future research should aim to address the identified limitations by including longitudinal designs, detailed pain characterization based on site and interventions, and direct assessments of relevant physiological factors. Additionally, exploring the role of frailty and other mediators, such as psychological well-being and medication use, will provide a more comprehensive understanding of the multifactorial nature of falls in older adults with chronic pain. It would be beneficial for future studies to explore balance and postural control in older adults to better understand the associations between chronic pain and movement kinetics.

## Conclusion

Chronic pain was associated with higher reporting of subsequent falls, frailty, poor physical performance and lower perceived health in at-risk older adults. Managing pain and its functional consequences should be an integral part of fall prevention strategies in older adults. Longitudinal studies are required to validate the impact of chronic pain management on the future burden of falls, frailty, quality of life and fear of falling.

## Data Availability

The datasets used and/or analysed during the current study are available from the corresponding author on reasonable request.
